# A genome-wide analysis in cluster headache points to neprilysin and PACAP receptor gene variants

**DOI:** 10.1186/s10194-016-0705-y

**Published:** 2016-12-13

**Authors:** Elena Bacchelli, Maria Michela Cainazzo, Cinzia Cameli, Simona Guerzoni, Angela Martinelli, Michele Zoli, Elena Maestrini, Luigi Alberto Pini

**Affiliations:** 1 Department of Pharmacy and Biotechnology, University of Bologna, Via Selmi 3, 40126 Bologna, Italy; 2 Headache and Drug Abuse Unit, Policlinico Hospital, University of Modena and Reggio Emilia, Modena, Italy; 3 Present address: School of Medicine, University of St Andrews, St Andrews, UK; 4 Department of Biomedical, Metabolic and Neural Sciences, Center for Neuroscience and Neurotechnology, University of Modena and Reggio Emilia, Modena, Italy; 5 Center for Neuroscience and Neurotechnology, Policlinico Hospital, University of Modena and Reggio Emilia, Via del Pozzo 71, 41100 Modena, Italy

**Keywords:** Cluster headache, Association studies in genetics, Genome-Wide Association Study, Pituitary adenylate cyclase-activating polypeptide receptor (*ADCYAP1R1*), Membrane metalloendopeptidase (*MME*), Neprylisin, Missense mutation

## Abstract

**Background:**

Cluster Headache (CH) is a severe primary headache, with a poorly understood pathophysiology. Complex genetic factors are likely to play a role in CH etiology; however, no confirmed gene associations have been identified. The aim of this study is to identify genetic variants influencing risk to CH and to explore the potential pathogenic mechanisms.

**Methods:**

We have performed a genome-wide association study (GWAS) in a clinically well-defined cohort of 99 Italian patients with CH and in a control sample of 360 age-matched sigarette smoking healthy individuals, using the Infinium PsychArray (Illumina), which combines common highly-informative genome-wide tag SNPs and exonic SNPs. Genotype data were used to carry out a genome-wide single marker case-control association analysis using common SNPs, and a gene-based association analysis focussing on rare protein altering variants in 745 candidate genes with a putative role in CH.

**Results:**

Although no single variant showed statistically significant association at the genome-wide threshold, we identified an interesting suggestive association (*P* = 9.1 × 10^−6^) with a common variant of the PACAP receptor gene (*ADCYAP1R1*). Furthermore, gene-based analysis provided significant evidence of association (*P* = 2.5 × 10^−5^) for a rare potentially damaging missense variant in the *MME* gene, encoding for the membrane metallo-endopeptidase neprilysin.

**Conclusions:**

Our study represents the first genome-wide association study of common SNPs and rare exonic variants influencing risk for CH. The most interesting results implicate *ADCYAP1R1* and *MME* gene variants in CH susceptibility and point to a role for genes involved in pain processing. These findings provide new insights into the pathogenesis of CH that need further investigation and replication in larger CH samples.

**Electronic supplementary material:**

The online version of this article (doi:10.1186/s10194-016-0705-y) contains supplementary material, which is available to authorized users.

## Background

Cluster Headache (CH) is a primary headache with a mean prevalence of 0.1% among general population and a clear male predominance [1]. It is characterized by severe unilateral retro-orbital or fronto-temporal pain attacks, occurring even eight times a day and associated with homolateral autonomic signs [2]. Pain attacks show a circadian and seasonal rhythmicity and smoking is the most frequent life-time habit in CH patients with a prevalence over 80%.

The biology of CH is poorly understood. Current hypotheses are focused on vasomotor changes, inflammation, immune changes, autonomic system imbalance and hypothalamic dysfunction [3]. Twin and family studies have indicated the importance of genetic factors in CH [1]. Genetic predisposition seems to be complex, with the environment and genetic factors interacting together [1].

Association studies focussing on candidate genes have suggested a possible role for *HCRTR2* [4–6], not confirmed in a recent meta-analysis [7]. No associations or conflicting results were found for variants in *CACNA1A* [8], *NOS* [9], *MTHFR* [10], *PER3* [11], *SERPINA* [12], and *ADH4* [13, 14] genes.

Given the largely unknown pathophysiology of CH, we have undertaken a whole-genome approach to identify the underlying genetic determinants in a clinically well-defined cohort of 99 Italian patients with CH. A cohort of 360 healthy Italian individuals, composed of age-matched cigarette smokers, was used as a control sample. We have used the Infinium PsychArray (Illumina) [15], which combines both common highly-informative genome-wide tag single-nucleotide polymorphisms (SNPs) and exonic SNPs, with an enrichment in genetic variants associated with common psychiatric conditions. This approach thus offers greater potential for the identification of rare or common variants with phenotypic effects in CH.

## Methods

### Study samples

One hundred patients with CH were enrolled at the Division of Toxicology and Clinical Pharmacology, Headache Centre, University of Modena and Reggio Emilia (Italy). CH diagnosis was made following the ICHD-III beta criteria [2]. CH patients clinical assessment included sex, age, headache history (age of onset, duration of the active phase, highest number of clusters per day and per month, triggering factors, associated autonomic symptoms, correlation with cigarette smoking in the active phase), drug treatment history and smoking status (number of cigarettes per day, CPD, and Fagerström test for nicotine dependence, FTND [16]). The control sample consisted of 360 age-matched Italian smokers, not suffering from CH or migraine, recruited at the same centre as part of a study on the genetics of nicotine dependence (RF-2009‐1549619). All participants were of self-reported Italian ancestry and provided a written informed consent to participate. This study was approved by the local Ethical Committee and took place in observation of the declaration of Helsinki (protocol number 2224/2013).

### Genotyping and quality control procedures

DNA for genotyping was extracted from blood or saliva. Genotyping was performed on Illumina Infinium® PsychArray microarrays (Illumina, San Diego, California, USA) in two batches, the first using Illumina PsychArray-24 v1.0, the second using PsychArray-24 v1-1 array [15]. We limited our analysis to 566178 variants present on both versions of the array. Content for the Infinium PsychArray includes 265,000 proven tag SNPs found on the Infinium Core-24 BeadChip, 245,000 markers from the Infinium Exome-24 BeadChip, and 50,000 additional markers associated with common psychiatric disorders.

The clustering algorithm implemented in GenomeStudio was used to cluster the data. Array clustering and quality control (QC) was performed according to recommended guidelines [17] (see Additional file 1 for details). The overall data quality was high: only one control DNA sample failed QC thresholds (one additional CH sample was subsequently removed because of non-European ethnicity); 10 replicate pairs of samples undergoing genotyping were assessed for consistency and reproducibility of the assay and agreement of genotyping calls was 99.99% over all genotypes across these pairs. Principal component analysis (PCA) was performed using PLINK 1.9 [18] on SNPs with minor allele frequency (MAF) > 0.01, pruned for linkage disequilibrium (200 kb window size, r2 > 0.5). PCA plots were generated using the Genesis tool [19]. One outlying sample was identified and excluded from subsequent association analysis (Additional file 1: Figure S1). Relatedness among subjects was checked using PLINK –genome option. 458 samples (99 cases, 359 controls) and 556485 variants pass filters and QC, of these 211927 are monomorphic and were excluded from association analysis.

Sanger sequencing was used to screen the *MME* coding region in all CH affected individuals. Primers sequences are available from the authors upon request.

### Association analysis

Single marker SNP association analysis was performed using the PLINK v 1.9 software [18]. Basic case-control analysis was performed on 290505 SNPs that passed QC and MAF > 0.01 using Fisher’s exact test. Results from the association analysis were clumped according to *P*-value using PLINK. Each clump is represented by an index variant (that with the lowest *P*-value in the clump), and contains all SNPs with *P*-value < 0.01 in linkage disequilibrium with it (R^2^ > 0.5, within 250 kb of the index SNP). Logistic regression was performed using PLINK –logistic command. To correct for multiple testing we applied the Bonferroni correction (P < 1.7 × 10^−7^, corresponding to 0.05/number of analyzed SNPs). Quantile-quantile (Q-Q) plot for test statistics was performed using the R package; the regional association plot was created using LocusZoom [20].

Gene-based association analysis was conducted using the Sequence Kernel Association Test (SKAT) [21], which aggregates individual score test statistics of a set of SNPs and efficiently computes gene-level *p*-values. Post QC, non-monomorphic SNPs were mapped to RefSeq genes (downloaded from the UCSC Table Browser, GRCh37/hg19). SKAT analysis was restricted to a comprehensive collection of candidate genes with a plausible role in CH pathogenesis. The selection of candidate genes was based on Gene Ontology (GO) biological process annotations (http://geneontology.org/page/download-annotations). We selected a group of 1038 genes annotated with sixty-six GO terms implicated in pain perception and response, blood pressure regulation and vasodilation/vasoconstriction, circadian rhythms, alcohol metabolism, neuropeptide signalling, ion channels, nicotinic acetylcholine receptors, tumour necrosis factor (TNF) signalling. The complete list of candidate genes and GO annotations is listed in Additional file 1: Table S1. Gene-based analysis was conducted on all protein altering variants (non-synonymous and splicing variants) with MAF <0.05 within these candidate genes, for a total of 2568 variants in 745 candidate genes (Additional file 1: Table S2). These included 2497 missense, 11 frameshift, 40 stopgain, 3 stoploss, and 17 splicing variants. We used a Bonferroni correction based on the number of the evaluated genes to assess the significance of the gene-based test results (p < 6.7 × 10^−5^). Functional annotation of variants was performed using Polyphen-2 [22] and the Combined Annotation–Dependent Depletion method (CADD) [23]. CADD is a tool for scoring the deleteriousness of variants in the human genome by incorporating a variety of algorithms in an integrated score (C score).

## Results

After stringent quality control filtering and exclusion of 211927 monomorphic SNPs, 344558 high quality SNPs were retained for genetic association analysis for CH disease susceptibility. The population sample included 99 individuals with CH and 359 controls. Demographic and clinical characteristics are summarized in Table 1. We conducted genome-wide association tests for single SNPs, as well as gene-based tests focussing on a subset of rare protein altering variants (PAVs), which *a priori*, are more likely to impact on disease status.

**Table 1:** Sample characteristics

	Cluster headache patients	Controls
*N*	99	359
Gender (*N*,%)		
Male	83, 83.84%	204, 56.82%
Female	16, 16.16%	155, 43.18%
Age, ys (mean ± sd, range)	47.39 ± 13.12, 23–81	48.96 ± 11.91, 18–70
CH attacks		
Age at onset, ys (mean ± sd, range)	30.04 ± 12.65, 6–80	
Chronic (*N*, %)	14, 14.14%	
N attacks/year (mean ± sd, range)	1.29 ± 0.77, 0.17–4	
N attacks/day (mean ± sd, range)	2.85 ± 1.68, 1–9	
Smoking		
Ever smoking (*N*,%)	88, 88.89%	359, 100%
N cigarette/day (mean ± sd)	19.17 ± 10.83	21.77 ± 9.13
Fagerström test (mean ± sd); *N*	4.60 ± 2.54 (*N* = 72)	5.95 ± 2.21

### Genome wide association analysis

**Fig. 1:**
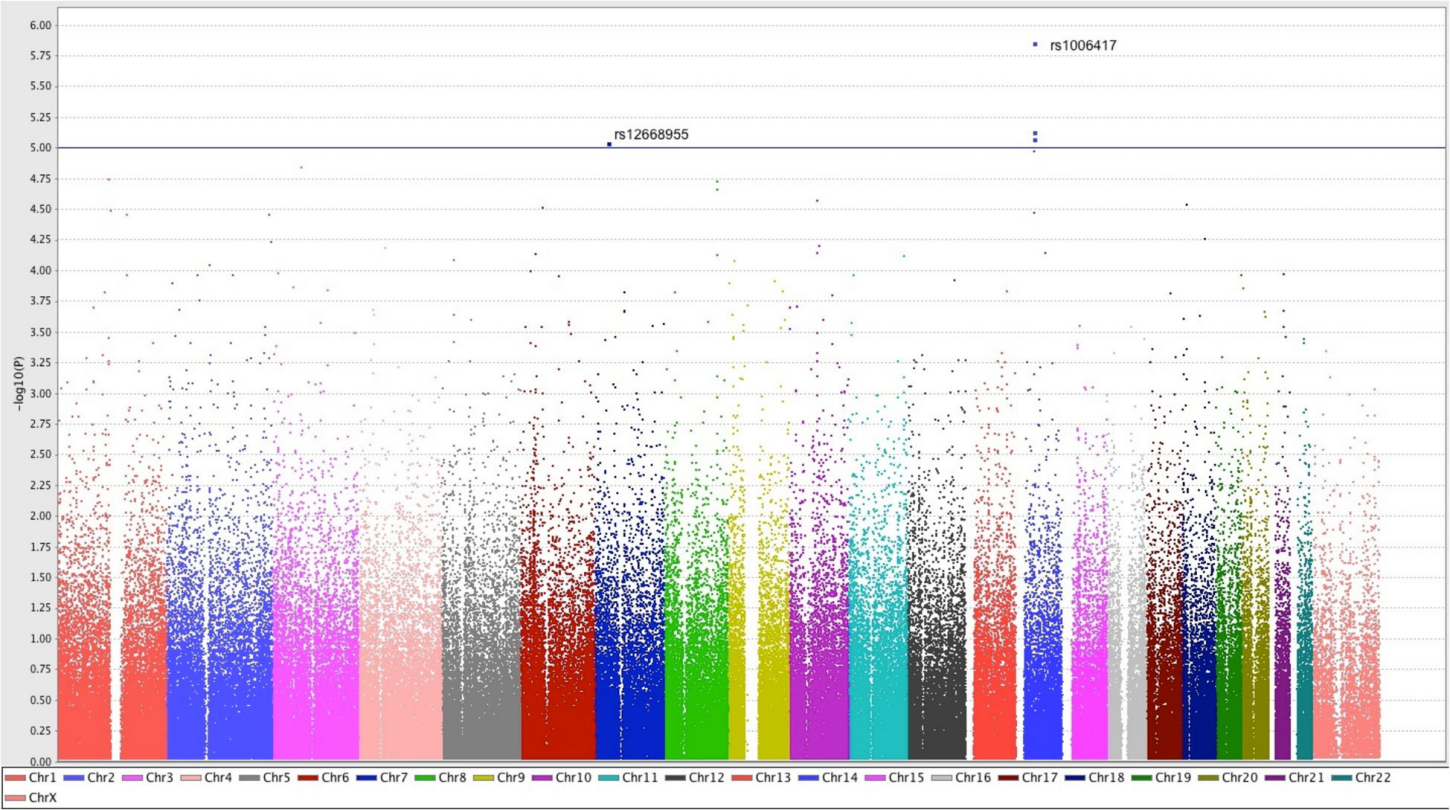
Manhattan plot of genome-wide association *P*-values. The y axis represents the -log_10_ Fisher’s test *P*-values; the horizontal line indicates the threshold for suggestive significance (*P* < 10^−5^)

Single marker case-control association analysis was performed on high quality SNPs with minor allele frequency (MAF) > 0.01 (290505 SNPs) in the study population, using the Fisher exact test (Additional file 1: Table S3). Calculation of the genomic inflation factor indicated no overall inflation of the genome-wide statistical results (λ =1), and the Q-Q plot displayed no global departure from the expected null distribution of *P*-values (Additional file 1: Figure S2). Both of these results indicate negligible inflation of the genome-wide association signals caused by population stratification. Overall association results are shown in the Manhattan plot (Fig. 1). Although no single variant showed statistically significant association at the genome-wide threshold (P < 1.7 × 10^−7^), we identified some suggestive associations (P < 10^−5^). Results of the top loci are shown in Table 2. A SNP cluster tagged by rs1006417 on chromosome 14 was the most significant hit (*P* = 1.4 × 10^−6^), located in a gene poor region with no known genes (Additional file 1: Figure S3). The second suggestive hit was for an intronic SNP in the ADCYAP receptor type I gene (*ADCYAP1R1)*, encoding for pituitary adenylate cyclase-activating peptide (PACAP) receptor (Additional file 1: Figure S3). Because CH patients are more frequently males, the male:female ratio differed in the cases and controls, we thus performed a logistic regression analysis with adjustment for sex as a covariate. This analysis confirmed the chromosome 14 locus as the most significant result while the significance of the *ADCYAP1R1* SNP was somewhat decreased (Additional file 1: Table S4), suggesting a possible sex effect at this locus. Adding adjustment for age and CPD to the logistic regression model provided similar results.

**Table 2:** Association analysis results of SNPs with suggestive genome-wide significance

Index SNP	Position	Other SNPs in clump	Gene	A 1	A 2	A1-F case/controls	A1-F 1000G_all (1000G_EUR)^a^	*P*-value	OR (95% CI)
rs1006417	14:41803291	rs1782180, rs1779522, rs1778408, rs11157219, rs715334, rs12433558		G	A	0.11/0.26	0.19 (0.19)	1.40 × 10^−6^	0.34 (0.21–0.55)
rs12668955	7:31116168		ADCYAP1R1	G	A	0.34/0.52	0.55 (0.50)	9.10 × 10^−6^	0.48 (0.34–0.66)
rs1495452	3:65834076		MAGI1	A	G	0.55/0.38	0.39 (0.45)	1.43 × 10^−5^	2.03 (1.48–2.79)
rs2182605	1:117463537		PTGFRN	G	A	0.32/0.50	0.51 (0.48)	1.80 × 10^−5^	0.49 (0.35–0.68)
rs16895584	8:122518935			A	G	0.24/0.04	0.070 (0.054)	1.85 × 10^−5^	3.50 (2.03–6.02)
rs6469999	8:122415207	rs13255877, rs7833779		A	G	0.31/0.17	0.46 (0.16)	2.16 × 10^−5^	2.23 (1.56–3.19)
rs1509957	10:64610718	rs224308, rs10822065		G	A	0.60/0.43	0.43 (0.49)	2.66 × 10^−5^	1.99 (1.44–2.74)

*Abbreviations*: *A1* minor allele in study sample, *A2* major allele, *A1-F* A1 frequency, *OR* odds ratio for minor allele, *CI* confidence interval

^a^Allele 1 frequency in 1000 Genomes phase 3, all populations (1000G_all) and european population (1000G_EUR)

Notably, our dataset did not provide support to previously reported putative association signals for CH in the *HCRTR2* and *ADH4* genes. None of the genotyped SNPs mapping to these genes, including the previously implicated SNPs rs2653349 (G1246A) in *HCRTR2* and rs1126671 in *ADH4*, reached a nominally significant *P*-value (Additional file 1: Table S3).

We also investigated a possible overlap with genetic susceptibility to migraine. A recent meta-analysis identified 45 independent confirmed SNPs that map to 38 distinct loci [24], of which 29 were directly genotyped or captured by a tag SNP in our study. Only rs9349379 in *PHACTR1* achieved a nominally significant *P* value (*P* = 0.034) (Additional file 1: Table S5).

Furthermore, given the relationship between CH and tobacco smoke, we hypothesized that genetic risk factors involved in smoking-related traits might also have a possible role in CH susceptibility. We inspected the *CHRNA5-CHRNA3-CHRNB4* cluster, the *CHRNA6*-*CHRNB3* cluster and *CHRNA4,* which are genes showing confirmed association to nicotine dependence and smoking behaviours [25–27]. However, no allelic imbalance was detected between CH cases and control smokers at any of the tested polymorphic variants (including the functional non-synonymous SNP rs16969968) (Additional file 1: Table S3). Given the availability of smoking measures in our sample, we also tested if an effect of these variants on smoking quantity (CPD) or nicotine dependence (FTND) was detectable in our sample. Linear regression analysis, with adjustment for sex, age, and CH case status, did not reveal any signal (data not shown).

### Gene based analysis

To increase power to identify disease causing variants, we restricted our analysis to rare (MAF < 0.05) PAVs mapping to a comprehensive collection of candidate genes associated to cellular processes with a plausible role in CH, including pain perception and response, vasodilation and vasoconstriction, circadian rhythms, alcohol metabolism, neuropeptide signalling, ion channels, nicotinic acetylcholine receptors, TNF signalling (Additional file 1: Table S1). A total of 2568 rare PAVs belonging to 745 candidate genes were included in gene-based analysis using SKAT [21], which produces a gene-level *P* value that indicates the degree of enrichment of rare variant associations for SNPs within a gene (Additional file 1: Table S1). The most significant results (nominal *P*-value < 0.001) are shown in Table 3. The membrane metalloendopeptidase gene (*MME)*, was the most significant gene with nominal *P* value of 2.5 × 10^−5^, which survived Bonferroni correction for multiple testing (*P* = 0.019). All the individual SNPs contributing to the gene-level tests (Table 3) had good genotyping cluster properties and genotyping rate > 99.9%. Interestingly, the most significant SNP (rs147564881) is a missense variant in *MME* (NM_000902.3:c.674G > C, p.G225A), which was found in 3.5% (7/99) CH patients and in none of 359 Italian controls. This variant is very rare in the general population (MAF = 0.1% in all 1000 Genome Project phase 3 individuals, 0.2% in 60,706 individuals of the Exome Aggregation Consortium ExAC), and has a possible damaging effect based on bioinformatic tools; the CADD PHRED-scaled C score is > 20, thus ranking in the top 1% of predicted deleteriousness of all possible single nucleotide variants [23]. The *MME* variants were confirmed by Sanger sequencing of the entire *MME* coding region in all CH patients; no additional rare variant was detected except for a synonymous SNP (rs200455903). Clinical details of the seven CH patients heterozygous for the c.674G > C (p.G225A) variant are shown in Additional file 1: Table S6; these subjects did not show significant differences in clinical aspects distinguishing them from the rest of the CH patient sample. Other nominal suggestive results (p < 0.01) were identified, including *NPY1R* (Neuropeptide Y receptor type 1), *BDKRB1* (B1 bradykinin receptor), *SLC5A3* (Sodium/myo-inositol cotransporter), *CCL26* (C-C motif chemokine 26) and *GRM1* (Metabotropic glutamate receptor 1) (Table 3).

**Table 3:** Results of SKAT gene-based tests in CH

SNP	SKAT *P*-value	Single SNPs *P*-value^a^	RA	RAF case/control	RAF 1000G_all (1000G_EUR)^b^	RAF in ExAC_all (ExAC_NFE)^c^	Function	PolyPhen-2 (Prediction)^d^	CADD score^e^
**Gene MME** (NM_000902.3)	**2.55** × **10** ^**−5**^								
rs61762319		0.42	G	0.030/0.021	0.0072(0.023)	0.0164(0.0226)	Missense c.22A > G (p.M8V)	0.524(P)	15.63
rs147564881		2.03 × 10^−5^	C	0.035/0	0.001(0.002)	0.0022(0.0033)	Missense c.674G > C (p.G225A)	1 (D)	21
rs61758194		1	A	0/0.0014	0.0002(0)	0.0021(0.0035)	Missense c.1033G > A (p.V345I)	0.001(B)	11.65
**Gene NPY1R** (NM_000909.5)	**1.18** × **10** ^**−3**^								
rs5578		3.80 × 10^−3^	C	0.040/0.0084	0.003(0.007)	0.0047(0.0067)	Missense c.1121A > C (p.K374T)	0.421(B)	12.96
rs78156188		1	A	0/0.0028	0.0004(0.002)	0.0006(0.001)	Missense c.332C > T (p.A111V)	0.023(B)	3.073
**Gene BDKRB1** (NM_000710.3)	**3.30** × **10** ^**−3**^								
rs143823168		1	C	0/0.0028	0.0018(0.003)	0.0024(0.0036)	Missense c.571C > G (p.L191V)	0.999(D)	9.214
rs45528332		9.23 × 10^−3^	A	0.030/0.0056	0.0036(0.004)	0.0062(0.0052)	Missense c.721G > A (p.G241R)	0.926(P)	9.661
**Gene SLC5A3** (NM_006933.6)	**4.94** × **10** ^**−3**^								
rs35707420		0.014	A	0.025/0.0042	0.0026(0.006)	0.0062(0.0087)	Missense c.1108G > A(p.V370M)	1(D)	18.46
rs199948762		0.39	G	0.0051/0.0014	.	0.000099 (0.0001)	Missense c.1671C > G(p.N557K)	0.001(B)	8.533
**Gene CCL26** (NM_006072.4)	**7.02** × **10** ^**−3**^								
rs41463245		0.022	A	0.020/0.0028	0.0036(0.016)	0.0086(0.0131)	Nonsense c.132G > A (p.W44X)	.	23.1
**Gene GRM1** (NM_000838.3)	**9.45** × **10** ^**−3**^								
rs41305288		0.017	A	0.035/0.0098	0.01(0.017)	0.0172(0.0185)	Missense c.2185C > A (p.P729T)	1(D)	19.24
rs362936		1	A	0.0051/0.0097	0.004(0.017)	0.0212(0.031)	Missense c.2651G > A (p.G884E)	0.998(D)	13.22
rs138759146		1	C	0/0.0014	.	0.0001(0.0002)	Missense c.2725A > C (p.M909L)	0.017(B)	1.328
rs2941		0.14	A	0.0051/0.022	0.0092(0.025)	0.0132(0.0182)	Missense c.2785G > A (p.V929I)	0.111(B)	18.3
rs79336287		0.071	A	0.015/0.0023	0.005(0.004)	0.0017(0.0013)	Missense c.3206C > T (p.P1069L)	0(B)	11.39

*Abbreviations*: *RA* rare allele, *RAF* rare allele frequency

^a^Single SNP Fisher's exact test *P*-value

^b^Rare allele frequency in 1000 Genomes all populations (1000G_all) and european populations (1000G_EUR)

^c^Rare allele frequency in Exome Aggregation Consortium (ExAC) Non-Finnish European (NFE) populations

^d^ HumDiv score; *B* benign, *P* possibly damaging, *D* probably damaging

^e^CADD v1.3, PHRED-like (-10*log10(rank/total)) scaled C-score

## Discussion

At present, the molecular genetic background of CH remains a largely unexplored field. Molecular genetic investigations of CH have been in part hampered by the complex nature of the disorder and have been confined to single candidate gene studies. GWAS offer a hypothesis-free alternative to candidate gene studies and have recently brought novel insights into the biological and genetic underpinnings of many complex traits, including migraine [24]. However, most GWAS have focussed on common variants in the general population (>5%) with a modest effect on risk, hence requiring very large sample sizes. The diffusion of exome sequencing technology provided evidence for the role of rare coding variants in the aetiology of several complex diseases, and a cost effective alternative to exome sequencing is provided by the recent development of “exome” genotyping arrays, which were created to genotype rare coding variants identified by exome sequencing of thousands of individuals [28]. Thus, in order to comprehensively investigate genetic factors influencing risk to CH, we have used the Illumina PsychArray [15] that allows testing genome-wide common variants as well as low frequency exonic variants: the combination of these two features results in a gain in power, given the unknown genetic architecture of CH. Genotype data were exploited to carry out single marker genome-wide association tests using common SNPs, as well as gene-based association analysis focussing on rare exonic variants in candidate genes with a putative role in CH.

The most interesting result stemming from genome-wide analysis was a suggestive association of CH with a common variant of the PACAP receptor gene (*ADCYAP1R1*); furthermore, gene-based analysis provided evidence for a rare variant of the *MME* gene. Both gene products are known to have a pivotal function in pain mechanisms, thus making these associations particularly stimulating.

PACAP is a peptide involved in pain-processing in animal models [29], and recently proposed to contribute to migraine pathogenesis [30]. Notably, PACAP administration in healthy subjects induces migraine-like headache [31]. PACAP, through PACAP receptor, induces activation of neurons in the superior salivatory nucleus, sphenopalatine and Gasser ganglion [32], that is thought to be a crucial event in CH pathophysiology, and higher PACAP plasma levels were detected during ictal phase than during inter-bout periods in CH patients [33].

*MME* encodes for a zinc-dependent plasma membrane endopeptidase, also known as neprilysin or neutral endopeptidase (NEP), that hydrolyses several extracellular peptides. They include peptides, such as substance P, neuropeptide Y, bradykinin, enkephalins, and atrial and brain natriuretic peptides [34], involved in the central regulation of pain, vasomotion, stress and sleep-wake rhythms as well as in the modulation of trigeminal nociceptive signals, whose alterations are thought to be implied in CH and to lead to the principal clinical features of this disease [3, 35, 36]. Interestingly, constitutive NEP knockout mice showed enhanced response to noxious thermal and chemical stimuli as well as thermal hyperalgesia and allodynia, and mechanical allodynia after chronic constriction injury of sciatic nerve [37]. The *MME* c.674G > C (p.G225A) missense variant was absent in our unaffected controls, but it is represented at low frequency in public control datasets (MAF = 0.02%); although the prevalence of primary headache in public datasets is unknown, this could reflect incomplete penetrance and the interaction with other genetic or environmental factors. Furthermore, rare loss-of-function and missense *MME* variants were recently associated to late onset dominant axonal polyneuropathies (Charcot-Marie-Tooth Neuropathy Type 2, CMT2; spinocerebellar ataxia with neuropathy, SCA43) [38, 39] and to a rare autosomal recessive variant of CMT2 [40]; none of the neuropathy patients described in these studies was reported to have migraine or CH symptoms, however individuals with MME mutations were reported to experience painful sensations [38]. Given the variety of NEP target peptides and their wide expression pattern, it is plausible that *MME* deleterious variants may contribute to the aetiology of different neurological conditions, and further studies are needed to identify the mechanisms underlying the phenotypic differences. We can speculate that while severe, very rare deleterious mutations lead to axonal polyneuropathies, the more prevalent c.674G > C (p.G225A) missense variant might have a milder damaging effect, increasing CH susceptibility by interaction with other environmental or genetic factors.

## Conclusions

In conclusion, our work provides the first evidence that genetic variants in genes involved in pain processing could confer susceptibility to CH. A major limitation in our study is the relatively small sample size, hence further investigation in larger cohorts is warranted in order to confirm our initial association results.

## Additional file

Additional file 1: Supplemental Methods. **Table S1.** list of selected candidate genes, GO terms annotations, number of rare protein altering variants (PAV) contained in each gene, and gene-level *P*-values obtained from SKAT analysis. **Table S2.** list of rare PAV (MAF < 0.05) identified in CH cases and controls in 745 candidate genes. **Table S3.** *P*-values for single marker *P*-values of all tested SNPs. **Table S4.** logistic regression analysis. *P*-values for additive effect of SNPs controlling for sex as a covariate (P sex), and for sex, age and cigarettes per day (P sex, age, CPD). **Table S5.** Association analysis in CH cases and controls for significant migraine susceptibility SNPs emerged in meta-analysis. **Figure S1.** Principal component analysis (PCA) plot. **Figure S2.** Quantile-quantile (Q-Q) plot of Fisher’s exact test *P*-values for association with cluster headache (CH). **Figure S3.** Local association plots for chromosome 14 locus **(A)** and the chromosome 7 locus **(B)** [17, 18, 24, 41]. (ZIP 6963 kb)

## Abbreviations

*ADCYAP1R1*: ADCYAP receptor type I gene
*BDKRB1*: B1 bradykinin receptor
CADD: Combined Annotation–Dependent Depletion
*CCL26*: C-C motif chemokine 26
CH: Cluster Headache
CMT2: Charcot-Marie-Tooth Neuropathy Type 2
CPD: Cigarettes per day
ExAC: Exome Aggregation Consortium
FTND: Fagerström test for nicotine dependence
GO: Gene Ontology
*GRM1*: Metabotropic glutamate receptor 1
GWAS: Genome-wide association study
MAF: Minor allele frequency
*MME*: Membrane metalloendopeptidase gene
NEP: Neutral endopeptidase
*NPY1R*: Neuropeptide Y receptor type 1
PACAP: Pituitary adenylate cyclase-activating peptide
PAV: Protein altering variant
PCA: Principal component analysis
QC: Quality control
Q-Q: Quantile-quantile
SCA43: Spinocerebellar ataxia with neuropathy
SKAT: Sequence Kernel Association Test
*SLC5A3*: Sodium/myo-inositol cotransporter
SNP: Single-nucleotide polymorphism
TNF: Tumour necrosis factor
