# Supplementary material for: A genome-wide analysis in cluster headache points to neprilysin and PACAP receptor gene variants
Source: J Headache Pain. 2016 Dec 13;17(1):114. doi: 10.1186/s10194-016-0705-y (PMC5153392; doi:10.1186/s10194-016-0705-y)
Supplement: Additional file 1: — Supplemental Methods. Table S1. list of selected candidate genes, GO terms annotations, number of rare protein altering variants (PAV) contained in each gene, and gene-level P-values obtained from SKAT analysis. Table S2. list of rare PAV (MAF < 0.05) identified in CH cases and controls in 745 candidate genes. Table S3. P-values for single marker P-values of all tested SNPs. Table S4. logistic regression analysis. P-values for additive effect of SNPs controlling for sex as a covariate (P sex), and for sex, age and cigarettes per day (P sex, age, CPD). Table S5. Association analysis in CH cases and controls for significant migraine susceptibility SNPs emerged in meta-analysis. Figure S1. Principal component analysis (PCA) plot. Figure S2. Quantile-quantile (Q-Q) plot of Fisher’s exact test P-values for association with cluster headache (CH). Figure S3. Local association plots for chromosome 14 locus (A) and the chromosome 7 locus (B) [17, 18, 24, 41]. (ZIP 6963 kb) [file 10194_2016_705_MOESM1_ESM.zip › Appendix_e-1.docx]

**Supplemental Methods**

Genotyping array clustering and quality control.

GenomeStudio was used to evaluate all genotypes using a quantitative genotype quality score called GenCall (GC) score, ranging from 0 to 1 with 1 being the best. The GC cutoff was set at 0.15, and the sample genotyping success rate cutoff was 95%. Based on these cutoff we We manually edited or zeroed all SNPs according to several GenomeStudio QC parameters, as recommended^17^: GenTrain score <0.6, low Cluster Separation values, low Call Frequency, low or high “AB R Mean” values (mean normalized intensity of the heterozygote cluster), low or high “AB T Mean” values (mean of the theta values of the heterozygote cluster), low or high “Het Excess” (indicator of the quantity of excess heterozygote calls relative to expectations based on Hardy-Weinberg Equilibrium) and very low minor allele frequency (MAF), given that very rare SNPs could be mis-called by the GenCall algorithm. Probes mapping as “0” (no position), “Y” (Y chromosome) and MT (mtDNA) were excluded.

After manual editing, genotypes were exported to the PLINK 1.9 software^18,41^ for additional data cleaning, to remove SNPs deviating from Hardy–Weinberg equilibrium (p<0.001), SNPs with missing genotyping rate >10% and markers with heterozygous haploid genotypes on the X.

**List of Supplemental Tables and Figures**

**Table e-1:** list of selected candidate genes, GO terms annotations, number of rare protein altering variants (PAV) contained in each gene, and gene-level P-values obtained from SKAT analysis.

**Table e-2:** list of rare PAV (MAF < 0.05) identified in CH cases and controls in 745 candidate genes

**Table e-3:** P-values for single marker P-values of all tested SNPs

**Table e-4:** logistic regression analysis. P-values for additive effect of SNPs controlling for sex as a covariate (P sex), and for sex, age and cigarettes per day (P sex, age, CPD).

**Table e-5:** Association analysis in CH cases and controls for significant migraine susceptibility SNPs emerged in meta-analysis ^24^

**Figure e-1:** Principal component analysis (PCA) plot

**Figure e-2:** Quantile-quantile (Q-Q) plot of Fisher’s exact test P-values for association with cluster headache (CH).

**Figure e-3**: Local association plots for chromosome 14 locus **(A)** and the chromosome 7 locus **(B)**
