# Supplementary material for: A genome-wide analysis in cluster headache points to neprilysin and PACAP receptor gene variants
Source: J Headache Pain. 2016 Dec 13;17(1):114. doi: 10.1186/s10194-016-0705-y (PMC5153392; doi:10.1186/s10194-016-0705-y)
Supplement: Additional file 1: — Supplemental Methods. Table S1. list of selected candidate genes, GO terms annotations, number of rare protein altering variants (PAV) contained in each gene, and gene-level P-values obtained from SKAT analysis. Table S2. list of rare PAV (MAF < 0.05) identified in CH cases and controls in 745 candidate genes. Table S3. P-values for single marker P-values of all tested SNPs. Table S4. logistic regression analysis. P-values for additive effect of SNPs controlling for sex as a covariate (P sex), and for sex, age and cigarettes per day (P sex, age, CPD). Table S5. Association analysis in CH cases and controls for significant migraine susceptibility SNPs emerged in meta-analysis. Figure S1. Principal component analysis (PCA) plot. Figure S2. Quantile-quantile (Q-Q) plot of Fisher’s exact test P-values for association with cluster headache (CH). Figure S3. Local association plots for chromosome 14 locus (A) and the chromosome 7 locus (B) [17, 18, 24, 41]. (ZIP 6963 kb) [file 10194_2016_705_MOESM1_ESM.zip › Supplemental Figures.pdf]

Figure e-1: Principal component analysis (PCA) plot.

The red arrow indicates the outlying sample excluded from genetic analysis.

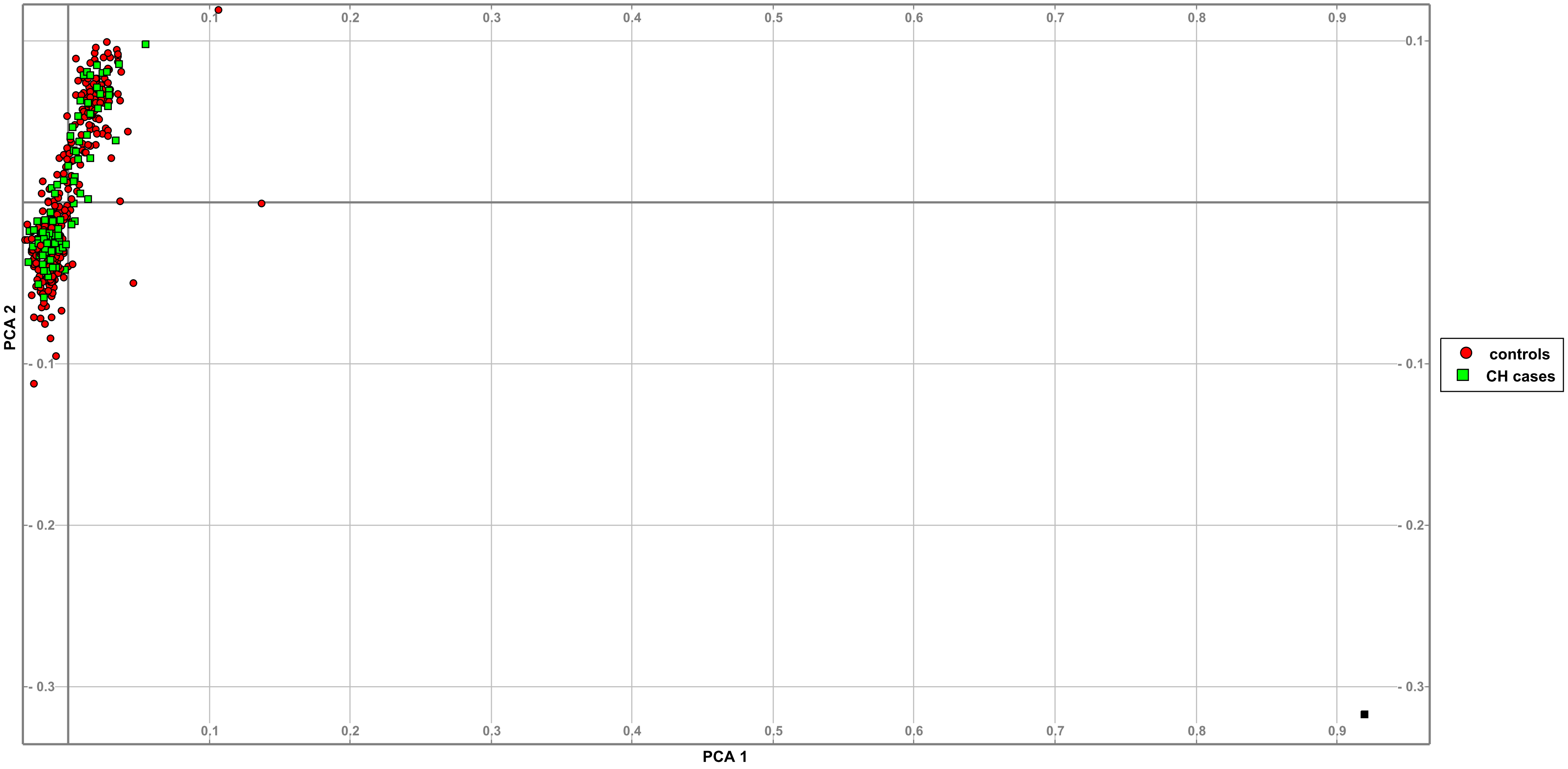

Figure e-2: Quantile-quantile (Q-Q) plot of Fisher's exact test P-values for association with cluster headache (CH).
X-axis shows P-values expected under the null distribution; Y-axis shows observed data. Grey region shows 95% confidence interval.

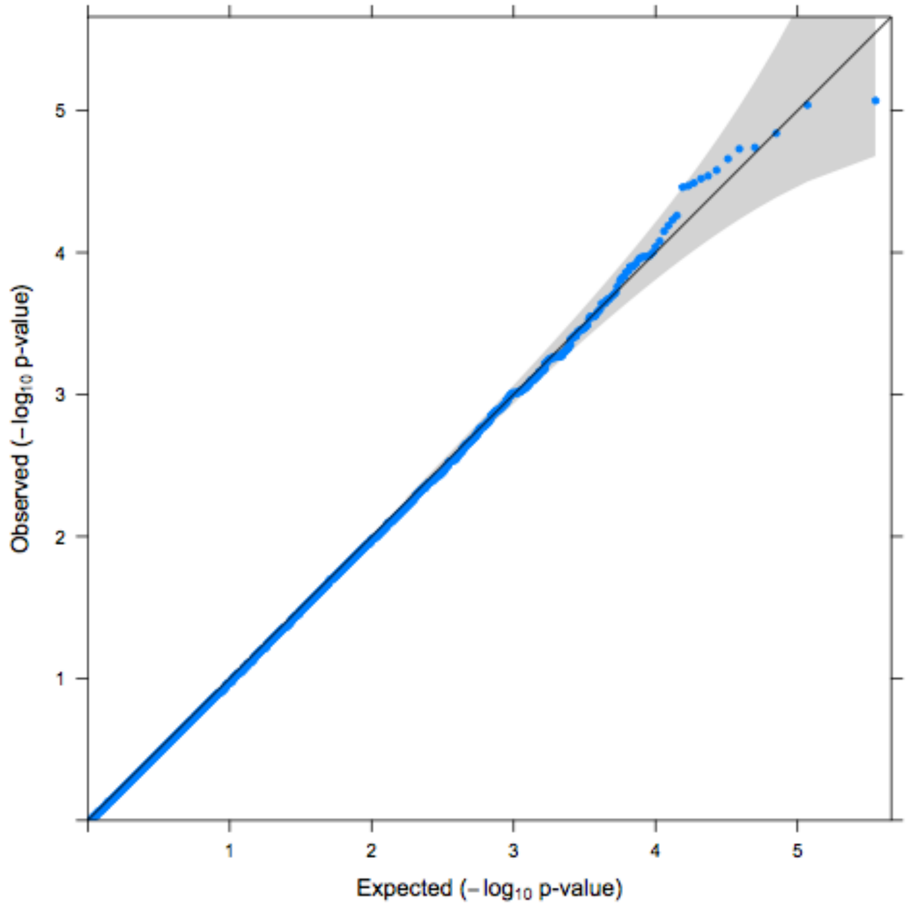

Figure e-3: Local association plots for chromosome 14 locus **(A)** and the chromosome 7 locus **(B)**
Association results are shown for genotyped SNPs, colored according to their level of linkage disequilibrium (LD) of the each SNP with the index SNP (purple diamond). The blue line shows the estimated recombination rates from the 1000 Genomes Project November 2014 release. The genomic locations of genes are shown below the plot based on GRCh37/hg19 assembly.

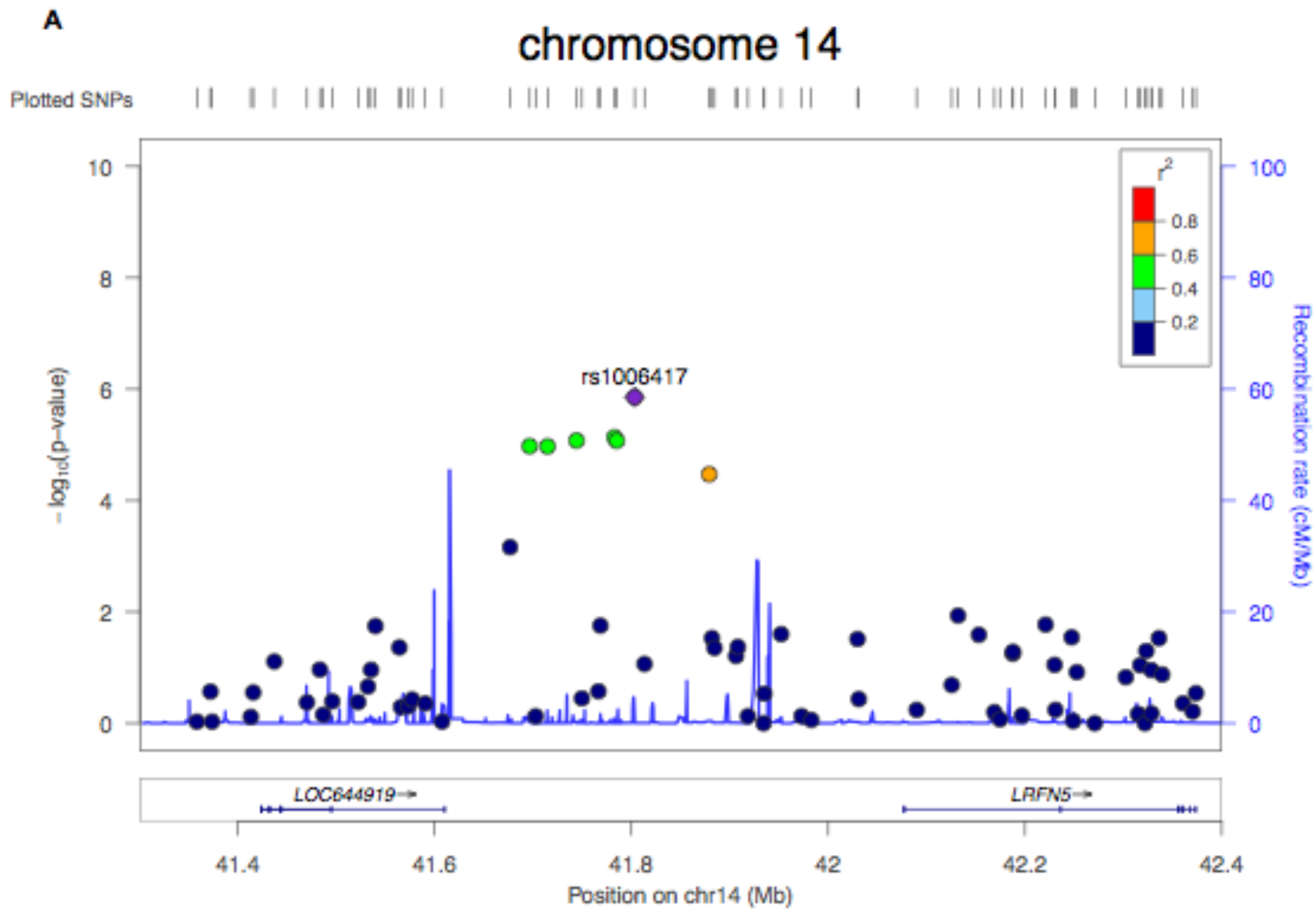

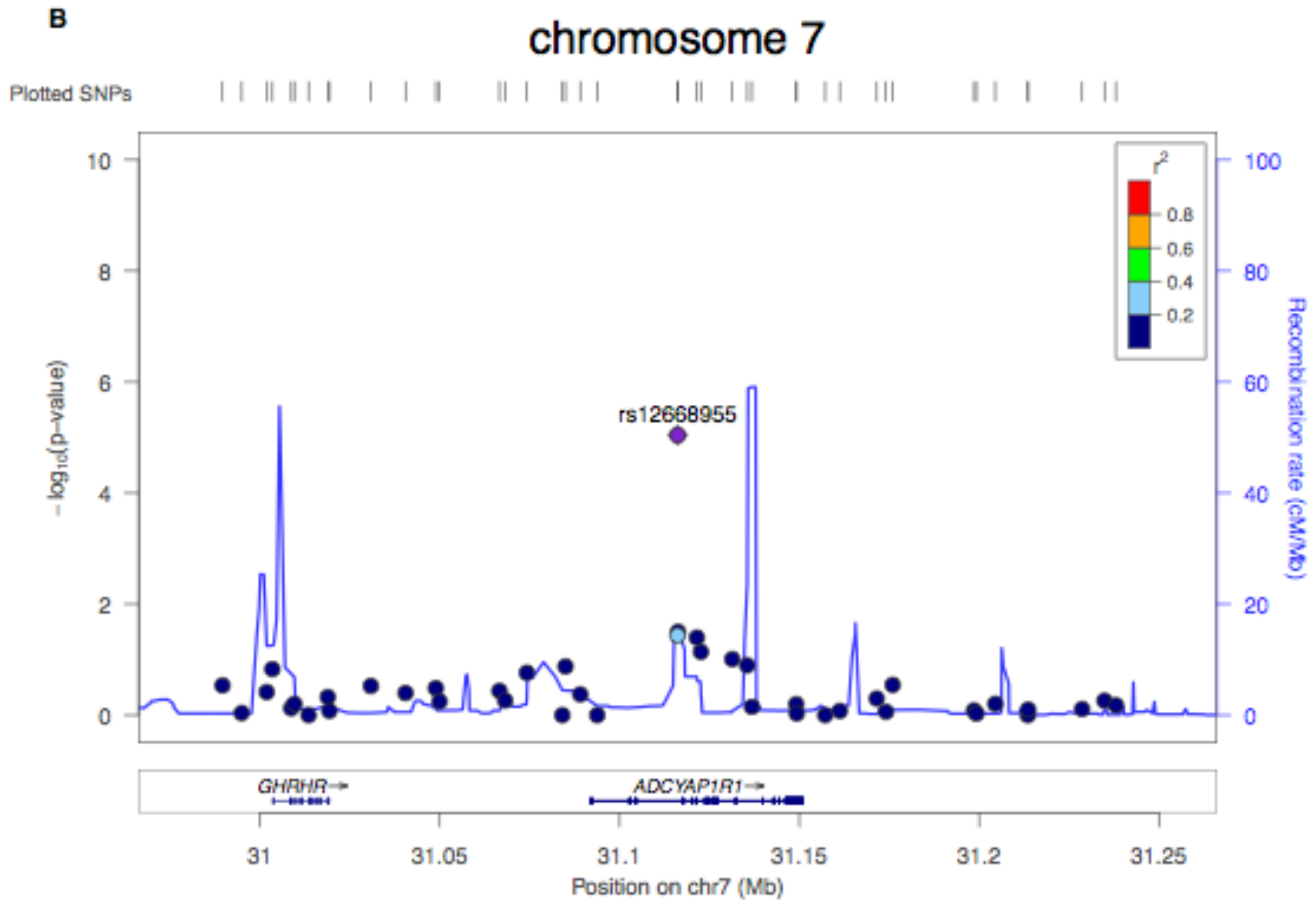
B
chromosome 7
Plotted SNPs
rs12668955
$-\log_{10}$(p-value)
Recombination rate (cM/Mb)
r^2
0.8
0.6
0.4
0.2
GHRHR
ADCYAP1R1
Position on chr7 (Mb)
31
31.05
31.1
31.15
31.2
31.25
0
2
4
6
8
10
20
40
60
80
100
